# GlycoForm and Glycologue: two software applications for the rapid construction and display of *N*-glycans from mammalian sources

**DOI:** 10.1186/1756-0500-3-173

**Published:** 2010-06-18

**Authors:** Andrew G McDonald, Keith F Tipton, Corné JM Stroop, Gavin P Davey

**Affiliations:** 1 School of Biochemistry and Immunology, Trinity College, Dublin 2, Ireland; 2 National Institute of Bioprocessing Research and Training, Belfield, Dublin 4, Ireland; 3 Merck, Sharp & Dohme, Oss, Netherlands

## Abstract

**Background:**

The display of *N*-glycan carbohydrate structures is an essential part of glycoinformatics. Several tools exist for building such structures graphically, by selecting from a palette of symbols or sugar names, or else by specifying a structure in one of the chemical naming schemes currently available.

**Findings:**

In the present work we present two tools for displaying *N*-glycans found in the mammalian CHO (Chinese hamster ovary) cell line, both of which take as input a 9-digit identifier that uniquely defines each structure. The first of these, GlycoForm, is designed to display a single structure automatically from an identifier entered by the user. The display is updated in real time, using symbols for the sugar residues, or in text-only form. Structures can be added to a library, which is recorded in a preference file and loaded automatically at start. Individual structures can be saved in a variety of bitmap image formats. The second program, Glycologue, reads a file containing columnar data of nine-digit codes, which can be displayed on-screen and printed at high resolution.

**Conclusion:**

A key advantage of both programs is the speed and facility with which carbohydrate structures can be drawn. It is anticipated that these programs will be useful to glycobiologists, systems biologists and biotechnologists interested in *N*-glycosylation systems in mammalian cells.

## Background

The explosion of interest in glycobiology in recent years, and the complex nature of its subject matter, have led directly to the requirement for bioinformatics tools specific to the needs of those researchers investigating the modelling of glycosylation, the carbohydrate content of glycoproteins, or the recombinant engineering of specific glycoforms in bioprocessing systems.

While bioinformatics tools specific to glycobiology are relatively few in number at present, applications for the construction and display of branched carbohydrate structures are beginning to appear. LiGraph, a web-based application based on the LINUCS linear notation [1], is able to draw JPG and SVG images from a linear string representation of a sugar. LiGraph supports a wide variety of symbol formats, including the Heidelberg, Tokyo, Consortium for Functional Glycomics (CFG) and Oxford (UOXF) notations. A more recent example is the GlycanBuilder [2], a Java tool that allows the user to draw *N*- or *O*-glycans using a wide range of core structures. GlycanBuilder also exports its glycan structures in a wide variety of bitmap and vector image formats. KegDraw [3], which is a powerful tool not only for drawing glycans, but chemical structures generally, allows the user to draw the structure of a glycan using a specialized tool palette and then query the KEGG GLYCAN database for similar structures.

**Figure 1:**
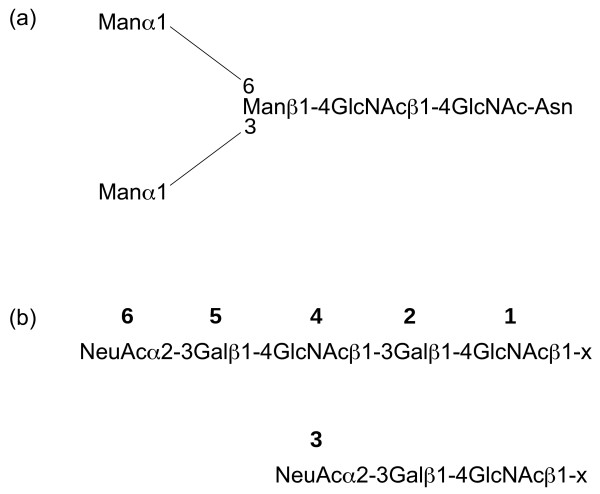
**The *N*-glycan encoding system of GlycoForm and Glycologue**. (a) The trimannosyl core structure, Man3, with the numeric value 300000000. (b) Extension levels of a branch attached to one of the exposed mannose residues of the core structure. The value of "x" is either 6 or 2 when a branch occurs at either of these two positions on the "upper" (α-1,6) mannose; "x" takes the values 4 or 2 at those positions on the "lower" (α-1,3) mannose. The extension level of a branch is encoded as a single digit from **0-6**. Extension levels **3 **and **6 **signify a *N*-acetylneuraminate (sialic acid) residue α-2,3-linked to galactose.

In this paper we present two new software applications for displaying *N*-glycans in mammalian cells: GlycoForm and Glycologue. Rather than adopting the palette-based approach of KegDraw and GlycanBuilder, both GlycoForm and Glycologue take as input the nine-digit identifiers devised by Krambeck and Betenbaugh [4]. The encoding system assumes the presence of a pentasaccharide, trimannosyl, core structure attached to an asparagine amino acid residue of a peptide or protein, as shown in Fig. 1(a), and can express a large number of high-mannose, hybrid and complex *N*-glycans. The first digit of the nine-digit code is the total number of mannose (Man) residues in the *N*-glycan, including those of the core structure; digit 2, the presence or absence of a fucosyl (Fuc) residue on the trimannosyl core structure; digit 3, the presence or absence of a bisecting *N*-acetyl glucosamine (GlcNAc) residue; digits 4-7 indicate the presence and composition of up to four antenna, attached to the α1-3 and α1-6 linked mannose residues of the trimannosyl core; digit 8, the number of galactosyl (Gal) units; digit 9, the number of *N*-acetylneuraminic acid (NeuAc) residues, of which can be up to four. The encoding system is summarised in Table 1. The numbering scheme of digits 4-7 is described in more detail in Fig. 1(b). GlycoForm draws a single structure at a time, whereas Glycologue can display multiple structures from a list of codes supplied by the user as a text file. Table 2 shows the enzymes [5] accounted for in the current encoding scheme. Since the enzymes listed are specific for certain bond types, the enzyme name reflects this. For example, N-acetyllactosaminide α-2,3-sialyltransferase catalyses the addition of an α-2,3-linked NeuAc residue to the galactose of an N-acetyllactosamide (Gal-GlcNAc) group.

**Table 1:** The N-glycan encoding system [4].

Digit	Allowed values	Meaning
1	3-6	Number of mannose residues
2	0 or 1	Core fucose
3	0 or 1	Bisecting GlcNAc
4	0-6	Extension level of branch 1
5	0-6	Extension level of branch 2
6	0-6	Extension level of branch 3
7	0-6	Extension level of branch 4
8	0-8	Number of galactose residues
9	0-4	Number of *N*-acetylneuraminic acid residues

**Table 2:** Glycosynthetic enzymes typically present in mammalian systems [5].

EC number	Enzyme name
2.4.1.38	β-*N*-acetylglucosaminylglycopeptide β-1,4-galactosyltransferase
2.4.1.68	glycoprotein 6-α-L-fucosyltransferase
2.4.1.101	α-1,3-mannosyl-glycoprotein 2-β-*N*-acetylglucosaminyltransferase
2.4.1.143	α-1,6-mannosyl-glycoprotein 2-β-*N*-acetylglucosaminyltransferase
2.4.1.144	β-1,4-mannosyl-glycoprotein 4-β-*N*-acetylglucosaminyltransferase
2.4.1.145	α-1,3-mannosyl-glycoprotein 4-β-*N*-acetylglucosaminyltransferase
2.4.1.149	*N*-acetyllactosaminide β-1,3-*N*-acetylglucosaminyltransferase
2.4.1.155	α-1,6-mannosyl-glycoprotein 6-β-*N*-acetylglucosaminyltransferase
2.4.99.6	*N*-acetyllactosaminide α-2,3-sialyltransferase
3.2.1.113	mannosyl-oligosaccharide 1,2-α-mannosidase
3.2.1.114	mannosyl-oligosaccharide 1,3-1,6-α-mannosidase

## Implementation

GlycoForm and Glycologue are written in the object-oriented language, REALbasic [6] and distributed as binary executable files for Windows, Mac OS X and Linux x86, and are freely available for download [7]. The Mac OS X version is packaged as a Universal Binary, which will run on both Intel- and PowerPC-based Macintoshes. The source code is also available from the applications' website.

### GlycoForm

**Figure 2:**
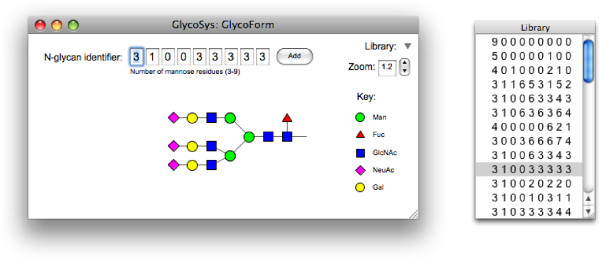
**GlycoForm output, showing the *N*-glycan structure with code 3 1 0 0 3 3 3 3 3 in CFG symbols**. Clicking on any digit in the GlycoForm code reveals the Krambeck and Betenbaugh descriptor for that position (see Table 1). Screenshot of the Mac OS X version.

GlycoForm is a desktop tool for drawing single *N*-glycan structures. Its main window is comprised of three main regions: input, control and display. The input region, shown at the top of the main window in Fig. 2, has an array of edit fields, the first seven of which can be changed by the user. Beneath this array is shown a description of the digit that has the current focus. In addition to a textual description, which in the case of digits 4 through 7 also includes the linkage type, the range of admissible values for the field is shown in parentheses. On loading, the focus is placed in the edit field of the first digit. Pressing the Tab key, or a valid number on the keyboard, will result in the entry of that digit, with the focus passing to the next field in the array. Digits 8 and 9 are reevaluated automatically whenever any of the first seven digits changes; hence, the focus passes back to the first digit whenever digit 7 is changed.

The greater part of the main window is devoted to the display of the oligosaccharide. If the window is resized, the drawable region rescales to accommodate the change. The current value of the magnification factor is shown to the top right of the main window and can be adjusted by the user (see Fig. 2). The default rendering method is CFG colour notation, however, menu options exist, which allow the user to toggle between text and symbol methods, and to choose between that, CFG black-and-white and UOXF notations. When a symbol set is chosen, the key appropriate to that set is displayed to the right of the oligosaccharide structure.

GlycoForm takes advantage of the system-wide clipboard present in each of the supported operating systems. A timer control, which activates once every second, reads the contents of the clipboard and, if its contents are text, a regular expression is used to test for the presence of a valid identifier. When a valid match is found, the display region is updated automatically with new structure using the current rendering parameters. While strings containing nine digits are accepted, the last two digits are ignored, since only the first seven of digits are necessary to specify a structure uniquely. A single space between each digit present on the clipboard is permitted, but not required, and any number of flanking spaces is permissible. Other than digits and spaces no other characters are accepted as valid.

Identifiers can be added to a library by pressing the Add button at the end of the edit-field array. The identifiers are shown as a list in the Library window, a floating window that can be opened or closed by clicking a small triangle at the upper right of the main window (see Fig. 2). *N*-Glycan identifier codes are selected for display by left clicking. Right-clicking (or control-clicking, using the Macintosh's single-button mouse) on any identifier in the library invokes a contextual menu with options to copy it to the clipboard, or to remove it permanently from the list. GlycoForm saves the current library of codes to a preferences file, along with the current display preference, symbolic or text-only. The file, which is an XML document, is saved to hard disk automatically when the program terminates, to a location appropriate to the current operating system.

GlycoBase [8] is a relation database of 2-AB labelled *N*-glycans. GlycoForm parses the current glycan identifier to form the corresponding GlycoBase abbreviation, whose display can be toggled by the user via a menu item. Our implementation of the GlycoBase formalism does not yet handle *N*-acetyllactosamine (NLac) repeating units (branch extension levels 4-6; see Fig. 1(b)) and is therefore currently limited to structures with one galactose residue per branch, of the type shown in Fig. 2. Individual structures drawn by GlycoForm can be saved as image files either Portable Network Graphics (PNG), JPEG or Windows Bitmap (BMP) formats.

### Glycologue

Glycologue is a tool for displaying or printing multiple *N*-glycan structures simultaneously. By default, it reads the user's current GlycoForm library file, loading and displaying any structures found therein in a grid. Text files containing 9-digit identifiers, one per line, is also an option. On systems supporting drag and drop, it is possible to open such a text file by dropping its icon onto that of the application. The number of grid elements is variable, but is fixed at 22 rows and 13 columns for the drag and drop method. Structures are drawn one column at a time, top to bottom, starting at the left-hand side of the main window. A secondary window displays the current list of identifiers. Clicking on an identifier in the list will highlight the corresponding image in the main Glycologue window.

Blank lines in the input file produce blank cells in the output, a feature that can be used to advantage if a graphical comparison between two or more sets of *N*-glycans is desired. The method consists in constructing the superset of all Glycologue input files that are to be compared, then sorting all files in numerical order. The second step is to create code-files from each of the same length as the superset, but with blank lines where a code in the superset is missing from the subset. This is difficult to accomplish manually, but is made easier by means of a script. The output files generated by such a script can then be loaded into Glycologue, where each *N*-glycan will appear at the same cell location as its counterpart in all the other files, including the superset. An example script, written in Perl, is provided as supplementary material [see Additional file 1].

Printing to PDF is supported natively by Mac OS X, and to PostScript by most Linux implementations. Windows users can use the free PDFCreator [9] printer driver to output as PDF. Being vector images, arrays of structures printed to PDF files are resolution-independent, and can be read and manipulated by many existing graphics tools.

Glycologue displays in one of the symbol formats supported by its companion program, GlycoForm, selectable via a menu option. Text-only display is not provided as an option, because in most instances the text will be too small to be legible. The identifier code is shown under each *N*-glycan by default, but can be hidden at the behest of the user.

### Rendering

**Figure 3:**
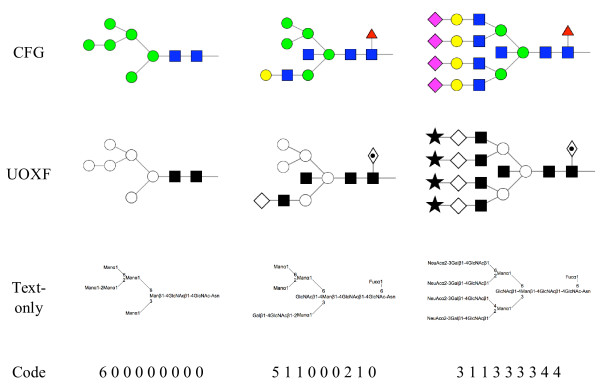
**Sample *N*-glycan structures depicted using both the CFG and UOXF symbolisms and in text-only form**. Oligomannose, hybrid and complex examples are shown. The corresponding identifier is displayed at the bottom of each column.

Both tools display N-linked oligosaccharide structures in right-to-left orientation, starting at the base Asn. The rendering code is maintained as a separate library, called Glycan, which helps to ensure parity of features whenever this code is modified. *N*-Glycans can be displayed in symbolic form using one of two symbol sets, CFG or UOXF, or in text-only form. (Currently, only the UOXF symbol set is supported, not the full set of linkage types and orientations of that system.) The output style is specified by means of a global integer variable. The *N*-glycan code is passed to the library as an integer array, to which also are passed the base coordinates of the structure and a magnification factor. Separate methods are used to draw closed geometric objects, such as circles, squares and diamonds. Depending on the symbol set selected by the user, the appropriate method is called for each residue in the oligosaccharide. Glycosidic linkages are represented as lines, the pen width being chosen as a function of the current magnification level. In symbol mode, residues are placed at a fixed distance from one another horizontally. Shapes representing monosaccharides are filled according to the active symbol set. Examples of output produced by the current rendering system are shown in Fig. 3.

## Results and Discussion

**Figure 4:**
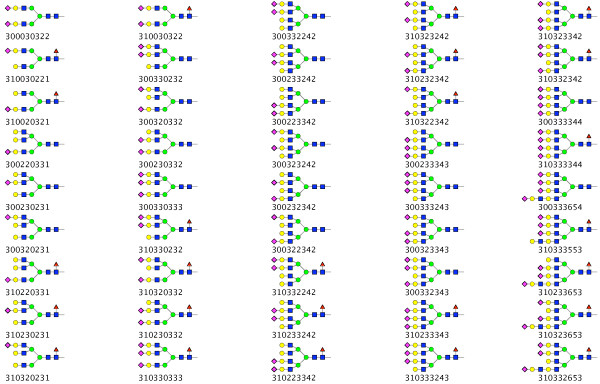
**Sample Glycologue output**. Shown are 45 *N*-glycan structures, with the Krambeck and Betenbaugh code printed beneath each; the structures are based on a study of recombinant human erythropoeitin expressed in Chinese hamster ovary cells [10].

An example of the output of Glycologue is shown in Fig. 4, where 45 *N*-glycans are displayed in CFG notation. The structures shown are those obtained by Restelli *et al. *[10] as part of a study of recombinant human erythropoeitin in Chinese hamster ovary cells. It should be noted that limitations of current methods of *N*-glycan analysis often prevent the full resolution of structures. For instance, the number of sialic acid residues may be determined more easily than the knowledge of their exact positions on each of the antennae. When the exact positions are unknown, a number of nine-digit identifiers may be written to cover every combination of sialylation pattern. For example, four numerical identifiers, 310333553, 310332653, 310323653 and 310233653, can be written for the structure A4G4FLac1S3. The software has also been used successfully to display other structures obtained by a variety of methods and from different mammalian sources. Data sets derived from two studies based on a CHO [11] and human choriocarcinoma [12] cell lines are available for download on the Glycologue website [7]. Despite the relative simplicity of the nine-digit encoding system, it can encompass a significant number of the most commonly encountered* N*-glycans. All the structures published by Hokke *et al. *[13] can be represented by the numerical identifiers, except for a small proportion that were determined to have Neu5Gc-capped antennae, where, as discussed below extension of the 9-sigit code would be required. The nine-digit system can also describe the majority of structures found in a caprine cell line [14] but further extension would be necessary to cope with the polyfucosylation reported in that work. Similarly, some, of the more complex structures discovered in a recent study of CHO-cell glycosylation mutants [15], such as poly-*N*-acetyllactosamine repeats, would necessitate additional numerical identifiers for display.

Of the symbols used by the two utilities, the CFG and UOXF formats each have their own merits: CFG is the most widely adopted, and has the support of one of the most popular glycobiology textbooks [16]; the UOXF notation, with its ability to describe different linkage type, is in theory able to encode more information in a single structure. Moreover, the symbols used by UOXF possess a higher degree of information content, for instance, in the use of a hexagon shape for hexose, and the use of solid fill to denote the *N*-acetylated variants of residues. A proposal for a new standard, which merges common elements of the CFG and UOXF symbolisms, has recently been presented [17].

Development of both tools is continuing. While the current implementations require the user to enter data in numeric form, it is hoped that input in other linear formats, such as the GlycoBase annotation system for 2AB-labelled *N*-glycans [18] or Linear Code [19], will be offered as options in a future release. While the display of structures in Glycologue is currently limited to a single window, this will be updated to allow for larger collections to be displayed within a scrolling window. It is further intended that the full complement of CFG and UOXF symbols, as well as the newly proposed standard referred to above, will be included; the UOXF variant will be supported by an updated rendering system to cope with new linkage types and residue positions, as well as expansion of the encoding system for both. In some instances, widening the range of allowable values of a digit might be sufficient. In the case of fucosylation, for instance, it would be possible to allow the second digit to possess values above 1, to encode fucosylation at different positions on the *N*-glycan, or with different linkage types. The present system was designed specifically for mammalian *N*-glycans but a similar approach could be used, with alterations to the encoding system, to accommodate the structures found in other species. Thus extra digits could be used for the additional residues, such as Xyl and Ara, which can occur in plant *N*-glycans [20], and for the pentaantennarity that can occur in certain ovomucoids from birds and fish [21,22].

## Conclusion

We have developed two new applications for the display of asparagine-linked oligosaccharides, both of which are freely available for use by the scientific community. The encoding system, which was initially developed specifically for the Chinese hamster ovary cell line, captures a subset of the *N*-glycan structures found in mammalian cells. A key advantage of the software is the rapidity with which it is possible to specify and display structures, the succinctness of the numeric encoding scheme permitting faster display and rendering of *N*-glycans than other utilities currently available.

## Availability and Requirements

Project home page: http://www.boxer.tcd.ie/gf

Operating system(s): Linux x86 (with GTK 2+); Mac OS × 10.4 or higher; Windows XP or higher

Programming langauge: REALbasic

License: Freeware

## Competing interests

The authors declare that they have no competing interests.

## Authors' contributions

AGM designed and wrote the software and drafted the manuscript. KFT and CJMS tested the tools and GPD oversaw the project. All authors read and approved the final manuscript.

## Supplementary Material

Additional file 1 **Glycologue input-file alignment utility**. a Perl script to align two sorted files containing 9-digit *N*-glycan identifiers. Click here for file
